# Generation of large coherent states by bang–bang control of a trapped-ion oscillator

**DOI:** 10.1038/ncomms11243

**Published:** 2016-04-05

**Authors:** J. Alonso, F. M. Leupold, Z. U. Solèr, M. Fadel, M. Marinelli, B. C. Keitch, V. Negnevitsky, J. P. Home

**Affiliations:** 1 Institute for Quantum Electronics, ETH Zürich, Otto-Stern-Weg 1, 8093 Zürich, Switzerland

## Abstract

Fast control of quantum systems is essential to make use of quantum properties before they degrade by decoherence. This is important for quantum-enhanced information processing, as well as for pushing quantum systems towards the boundary between quantum and classical physics. ‘Bang–bang' control attains the ultimate speed limit by making large changes to control fields much faster than the system can respond, but is often challenging to implement experimentally. Here we demonstrate bang–bang control of a trapped-ion oscillator using nanosecond switching of the trapping potentials. We perform controlled displacements with which we realize coherent states with up to 10,000 quanta of energy. We use these displaced states to verify the form of the ion-light interaction at high excitations far outside the usual regime of operation. These methods provide new possibilities for quantum-state manipulation and generation, alongside the potential for a significant increase in operational clock speed for trapped-ion quantum information processing.

## Abstract (web summary)

Fast control of quantum systems is essential to exploit their properties for information processing before they are lost to decoherence. Here the authors demonstrate the use of quasi instantaneous changes to the trapping potential of a trapped-ion to create highly-excited quantum coherent states.

## Graphical abstract

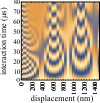

Coherent quantum control involves the manipulation and exploitation of high-purity quantum states, which constitute a fundamental resource for quantum information processing^1^. For quantum harmonic oscillators, experiments have demonstrated such control using confined optical and microwave fields^2^,^3^, and using the mechanical oscillations of single trapped ions^4^,^5^,^6^,^7^. Most of this work was performed in the adiabatic and resonant regimes, where control timescales are much slower than the system's natural frequency. ‘Bang–bang' control is the opposite extreme, in which timescales are so short that changes to the system can be considered to be instantaneous^8^,^9^,^10^. These techniques are commonly used to prolong the coherence of a quantum system. For trapped ions, bang–bang control has been performed using ultra-fast laser pulses to induce sudden momentum kicks along directions dependent on the internal electronic state of the ions^11^,^12^. However, optical fields interact with the electric dipole or quadrupole moment of the atom, and the oscillator states prepared in this way have thus far been limited to excitations of <20 quanta. Electric fields acting directly on the charge (electric monopole) can be used for achieving a strong-interaction regime. This is a key element for transporting trapped-ion qubits in a scalable quantum-computing architecture^13^,^14^, where operations have been performed on timescales comparable to the oscillator frequency^15^,^16^. In these examples the speed was limited by the bandwidth of the filtered control voltages applied to the electrodes.

Here, we demonstrate bang–bang control over trapped-ion harmonic oscillators, which we use to generate and characterize quantum states with up to 10,000 quanta. To achieve this, we wire several of the trap electrodes according to a new scheme: we connect each to the output of a switch which is placed in vacuum, close to the trap electrode. The two inputs to the switch are low-pass filtered analogue voltages, supplied from outside the vacuum system. The output is switched between these inputs based on the state of a digital signal (details can be found in the Methods section). For this set-up we measure switching times below 4 ns (ref. 17). This is much faster than the ion oscillation period, meaning that we can induce quasi-instantaneous changes to the oscillator states. Using these techniques, we map out the ion-light coupling strength for high excitations, and track the oscillation trajectory of a single ion throughout multiple oscillation cycles.

## Results

### Bang–bang generation of coherent states

**Figure 1:**
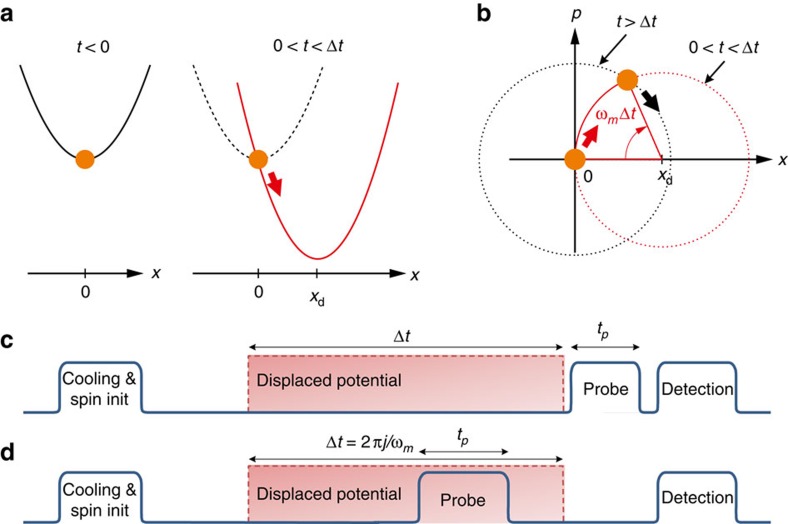
Experimental bang–bang sequences. (**a**) The ion is first cooled close to the ground state of motion in a harmonic trapping potential centred at *x*=0, and the internal electronic state is pumped to the ground-state |↓〉. At *t*=0 the potential center is suddenly displaced to *x*_d_ and the ion's motional state is the coherent state |−*α*_0_〉. (**b**) Evolution of the motional wave function in the position-momentum phase-space. After a time Δ*t* in the displaced potential, the trap centre is switched back to *x*=0, leaving the ion in a coherent state of size given by equation (1). (**c**) Sequence of laser pulses and potential displacements for monitoring the time evolution of coherent states. (**d**) Sequence for studying the light-matter interaction with highly excited states. In this sequence, Δ*t* is an integer number *j* of multiples of the oscillator period.

We prepare a scenario which is highly familiar in classical physics. Starting with a particle at rest at the bottom of a harmonic potential, we suddenly displace the latter by a distance *x*_d_ (Fig. 1a). For an instant, the particle finds itself at rest but in an excited state, and subsequently oscillates in the new potential. In our experiments, this situation is reproduced in the quantum regime. In the ideal case, the initial state is the quantum ground state |0〉, which is a minimum-uncertainty wave packet of root-mean-squared extension *x*_0_. The action of the sudden displacement produces a coherent state |−*α*_0_〉 with *α*_0_=*x*_d_/(2*x*_0_), which oscillates at a frequency *ω*_m_ while maintaining the form of the wave packet. Switching the potential minimum back to *x*=0 after a time Δ*t* leaves the oscillator in a coherent state of size


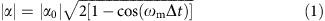


in the original potential (Fig. 1b). We verify the timing quality and reproducibility of our control through our ability to achieve |*α*|=0 after a full cycle of oscillation.

The trapped-ion oscillator we consider also has internal degrees of freedom, from which we can isolate a two-state pseudo-spin system (|↓〉, |↑〉) with transition frequency *ω*_0_. This transition can be coupled to the oscillator using electromagnetic radiation at frequency *ω*_L_=*ω*_0_+*sω*_m_. For integer *s*, resonant transitions can be induced on the *s*^th^ sideband between the states |↓〉 |*n*〉↔|↑〉 |*n*+*s*〉 with a Rabi frequency^18^


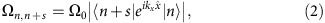


where Ω_0_ is a constant which contains the internal-state coupling strength and the electric field amplitude, *k*_*x*_ is the size of the radiation wavevector projected on the oscillation direction and |*n*〉 are the number states of the oscillator. For *k*_*x*_*x*_0_<<1 and at low excitations, a small parameter expansion of the exponential is sufficient to describe the resulting dynamics. This is the regime in which most quantum control experiments with trapped ions have been operated in the past. As a result, coherent control of pure quantum states has only been demonstrated for |*s*|≤2. With neutral atoms in a state-dependent standing wave, the relative strengths of excitation for a ground-state atom have been observed in a frequency spectrum containing resolved sidebands up to *s*=14 (ref. 19). In the work presented below, we map out the coherent excitations for 0≤*s*≤5, confirming the dependence of the light-atom interaction strength for excitations up to *n*≈10,000.

In our experiments, the oscillator is the axial motion of a single ^40^Ca^+^ ion confined in a radio-frequency trap with a frequency between *ω*_m_/(2*π*)≈2.35 MHz and 2.53 MHz, and corresponding values of *x*_0_∼7 nm. We use the dipole-forbidden transition at wavelength *λ*≈729 nm to define a pseudo-spin system between levels |↓〉≡|*L*=0, *J*=1/2, *M*_*J*_=−1/2〉 and |↑〉≡|*L*=2, *J*=5/2, *M*_*J*_=−5/2〉. The wavevector of the laser addressing this transition makes an angle *θ*≈45° with the motional direction, resulting in a Lamb-Dicke parameter *η*=*k*_*x*_*x*_0_ between 0.0439(1) and 0.0455(1). Each experimental sequence begins by initializing the trapped-ion oscillator close to its ground state using a combination of Doppler and electromagnetically induced-transparency cooling^20^. We measure a typical mean thermal excitation after cooling to be 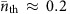.

**Figure 2:**
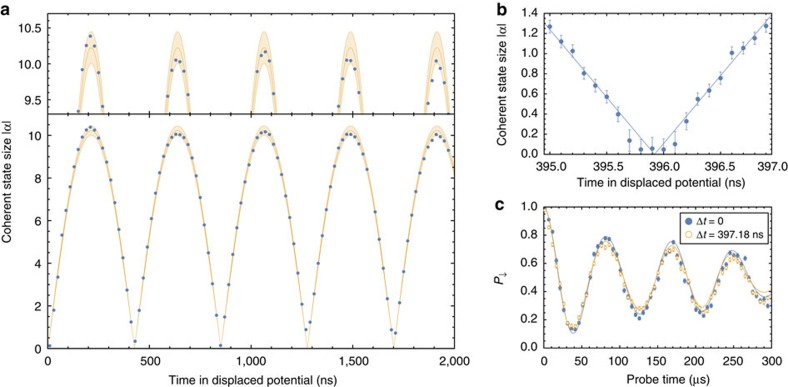
Coherent-state generation. (**a**) Plot of the final coherent state size |*α*| as a function of the evolution time Δ*t*. The states are prepared in the original potential by executing the experimental sequence in Fig. 1c. The solid orange line is a fit to the data using equation (1). The fluctuations in amplitude can be explained by drifts in the laser power over the time taken to acquire the data (shaded area, Methods section). (**b**) Sub-nanosecond resolution scan around one complete motional period (Δ*t*≈2*π*/*ω*_m_). This measurement was performed with *ω*_m_/(2*π*)≈2.53 MHz and corresponds to |*α*_0_|≈85. (**c**) Measured probability of finding the spin in the |↓〉 state as a function of the probe time using the *s*=1 sideband. Filled symbols correspond to the initially prepared state and open symbols after approximately one motional period. This measurement is independent of the previous. Here, *ω*_m_/(2*π*)≈2.52 MHz, 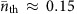 and |*α*_0_|≈85. Every data point consists of 1,000 measurements. Error bars are given as s.e.m. and assigned assuming quantum projection noise according to ref. 37.

In a first set of experiments we characterize our control using two successive displacements of the potential well (Fig. 1c). At *t*=0, we switch the centre of the potential from 0 to *x*_d_ (Methods), resulting in the ion being excited and subsequently oscillating in the displaced potential well. At *t*=Δ*t* we reverse this change, bringing the potential back to its original position. In the ideal case, the ion is then in a coherent state of size |*α*| given by equation (1). To read out the oscillator state, we apply an optical probe pulse of duration *t*_p_ resonant with the *s*^th^ sideband, followed by a detection of the ion's internal state using resonance fluorescence (Methods section). By repeating the experiment a large number of times for each value of *t*_p_, we obtain an estimate of the probability of finding the ion spin down as a function of time. This agrees well with the functional form


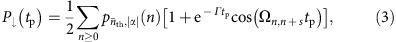


where 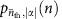 is the occupation of the *n*^th^ number state for a displaced thermal state with thermal mean quantum number 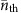 and displacement |*α*| (this distribution is given in the Methods section). The effects of spin and motional decoherence during the probe pulse are accounted for by an exponential decay at rate *Γ* (ref. 4). Experiments were performed for a range of values of Δ*t*, in each case taking data using the first sideband *s*=1 for 1.4 μs≤*t*_p_≤100 μs. For a single value of Δ*t*, the data do not allow the extraction of |*α*|, Ω_0_ and the decay parameter *Γ* independently. We therefore probe first the non-displaced state directly after cooling. Fitting using equation (3) with |*α*|=0 and 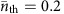, we determine Ω_0_/(2*π*)=181(1) kHz and *Γ*=2.2(3) ms^−1^. Error bars throughout this paper are given as s.e.m. These are then fixed in fits to the data for each value of Δ*t*, from which we extract the corresponding displacements |*α*|. The resulting values are plotted in Fig. 2a, and show clearly our ability to resolve the cycles of oscillation of the ion. Also shown is a fitted curve using equation (1), which allows us to determine the oscillation frequency of the ion *ω*_m_/(2*π*)=2.3505(6) MHz and amplitude *α*_0_=5.11(1), corresponding to *x*_d_=75.0(1) nm (more details of this analysis can be found in the Methods section). We observe in the results in Fig. 2a that the amplitude pertaining to each oscillation period of the ion appears to be different. We attribute this to drifts in the laser intensity at the ion, which result in shifts in the deduced value of |*α*| (this drift is illustrated by the bounding curves in Fig. 2a, see Methods section for further details).

To verify that the switching between potentials maintains coherence, we examine our ability to catch the ion in the ground state after a single cycle of oscillation. In an independent measurement we obtained data with Δ*t* spaced by 100 ps in the region around Δ*t*=2*π*/*ω*_m_ for *ω*_m_/(2*π*)≈2.53 MHz and an intermediate coherent state of |*α*_0_|=84.9(12). To faithfully return these states to the origin after a single trap cycle we need to trigger the experimental sequence from the phase of the radio-frequency trap drive. This can be attributed to a pseudopotential gradient along the axis of our trap^21^. The results are presented in Fig. 2b and show |*α*|=0.05(10) for Δ*t*≈2*π*/*ω*_m_. Figure 2c is a separate measurement with *ω*_m_/(2*π*)≈2.52 MHz, and provides a direct comparison between data for a non-displaced ion with 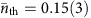, and one with the same starting temperature which has been displaced to |*α*_0_|=85.0(12) and returned one cycle later. A fit to the data for the latter gives 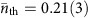 and consistent values for Ω_0_ and *Γ*.

### Light-matter interactions

**Figure 3:**
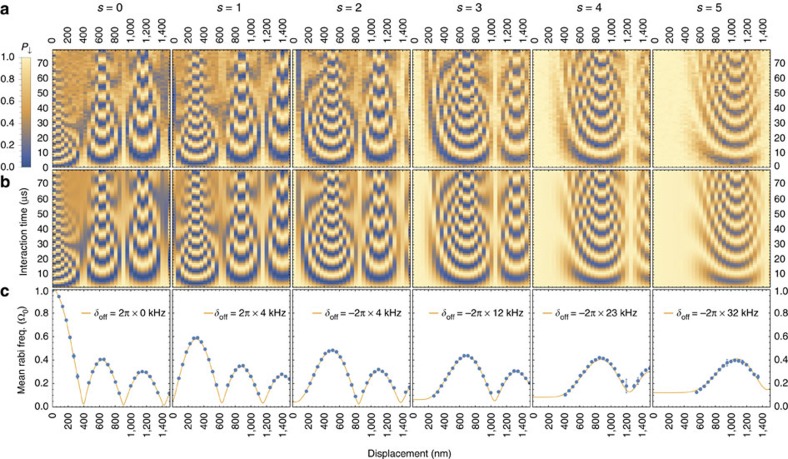
Light-atom interaction. (**a**) Measurement of the probability *P*_↓_ of finding the spin in the |↓〉 state as a function of the interaction time with light near-resonant with the *s*^th^ motional sideband and of the trap-potential displacement *x*_d_. The minimum pulse time is *t*_p_=1.4 μs, so for high Rabi frequencies, *P*_↓_ can differ from 1 even for the first point. (**b**) *P*_↓_ obtained using equation (3) with the parameters determined from fits. (**c**) Mean Rabi frequencies for displaced thermal states as a function of the potential displacement. The data points are given by the center of the Lorentzian curves which we fit to 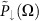, the Fourier transform of *P*_↓_(*t*_p_). Most error bars are hidden behind the points. The solid lines result from the theory model including AC Stark shifts and a detuning *δ*_off_ obtained from fitting the data for a given *s*.

Having characterized our control, we perform a second set of experiments to map out the interaction between the ion and the light field as a function of the motional excitation. In contrast to the work described above, we send the probe pulse to the ion while the potential is centred at *x*_d_. Subsequently, the potential well is returned to *x*=0, timed such that the ion completes an integer number of oscillation periods in the displaced well. In this way, we prevent the high oscillator excitation from affecting the internal state detection. Experimental measurements of *P*_↓_ are shown in Fig. 3a for sidebands between *s*=0 and *s*=5 and displacements up to *x*_d_=1.49(2) μm, which corresponds to |*α*_0_|=99.7(13) and 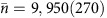 at a trap frequency of 2.35 MHz. For each value of *x*_d_, we probe *P*_↓_ using laser-pulse durations between 1.4 and 80 μs. To analyse these measurements, we start by determining Ω_0_/(2*π*)=204.3(1) kHz and 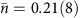 from measurements with the non-displaced state using the *s*=0 and *s*=1 sidebands, respectively. We then calibrate the displacement sizes using the data taken for the carrier transition *s*=0, fixing *Γ*=0 and floating only |*α*| in equation (3) (for further details, see Methods section). These calibrated values were then fixed, and data for each of the other sidebands was fitted as a full set using a model including motional-excitation-dependent AC Stark shifts due to nearby atomic transitions and a systematic detuning to account for miscalibration (Methods section). We find that the AC Stark shifts become relevant for *s*=4 and *s*=5 at high motional excitations, because the frequencies of these transitions are close to the seventh and sixth red motional sidebands, respectively, of an atomic transition, which is 25.74 MHz away from the driven transition. Two-dimensional plots showing the fit results are shown in Fig. 3b below the respective data sets.

To verify the predicted form of the matrix elements in equation (2), we take a Fourier transform of *P*_↓_(*t*_p_) for each value of *x*_d_. This gives us the Rabi frequency distribution, which is peaked due to the narrow distribution of number states that contribute significantly to the coherent state (Methods section). Figure 3c shows the mean Rabi frequency obtained from each Fourier transform versus the amplitude of the state. We overlay this with theoretical curves using equation (2), which include modifications for the offset detunings and AC Stark shifts. They show good agreement between experiment and theory over the full range of motional occupations. The minima in Rabi frequencies observed for each set of sideband data are separated by between 515 and 525 nm. These arise due to the large amplitude of the ion's motion, which spatially samples multiple wavelengths of the light. The separation between minima approaches the effective wavelength of the light as projected on the trap axis for *x*_d_>>*λ*. In our case this value is *λ*/(2cos*θ*)≈515 nm (for more details see Methods section). We note that although the *s*=5 sideband would take >1 minute to invert the spin if driven in the ground state, the modulation of the light due to the large ion oscillation allows us to do this in <10 μs.

## Discussion

The work presented has a number of possible applications for quantum information science. It may be used to speed up the transport of trapped-ion qubits in scalable quantum information processing^14^,^15^,^16^,^22^, where the transport would proceed by switching the position of the potential well to two positions *x*_d_ and 2*x*_d_ at times which are separated by Δ*t*=*π*/*ω*_m_ (ref. 17). This would result in the ion being ‘caught' in the ground state of the potential centred at 2*x*_d_, with the transport taking half a period of the ion's oscillation. Furthermore, we expect that large squeezed states could be achieved through sudden trap frequency changes, in this case separated by delays of one quarter of the trap period^17^. Bang–bang control routines based on sudden changes to the trap frequency have been proposed for suppressing motional decoherence effects^23^. Using ion chains, the ability to change the trapping potential on timescales fast compared with the characteristic frequencies of the interaction between ions has been proposed as a method for generating high levels of continuous-variable entanglement by means of the Coulomb interaction^24^,^25^,^26^. These protocols involve local squeezing of the mechanical motion of the ions by sudden changes to the curvature of the radial trapping potential. The Coulomb interaction leads to a de-localization of the squeezing, distributing it amongst all the ions in the chain and thereby entangling the mechanical motion of the localized ions.

## Methods

### Trap set-up

**Figure 4:**
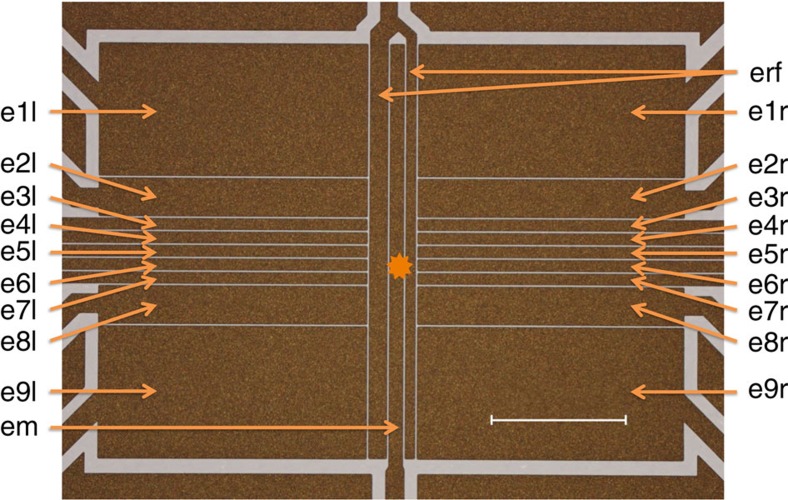
Surface-electrode trap. Linear Paul trap in final fabrication steps. Ions are trapped in the position denoted with a star, ≈50 μm from the trap surface. The length of the scale bar is 500 μm.

Our experiments are performed using a surface-electrode linear radio-frequency (rf) trap in a 5-wire asymmetric configuration^27^,^28^ (Fig. 4). The pseudo-potential null line is ≈50 μm above the trap chip. For these experiments the main trapping zone, denoted with a star in the figure, is between the pair of electrodes e6, closer to e6r than e6l due to the asymmetry in the width of the rf electrodes. The trap is driven with an rf amplitude of ≈100 V and a frequency of ≈93 MHz, leading to radial secular motion at ≈4 and ≈7 MHz at an axial frequency of *ω*_m_/(2*π*)≈2.5 MHz.

The trap is placed in a sealed chamber cooled down to ≈4 K. Cryogenic set-ups yield improved vacuum over room-temperature experiments^29^, resulting in longer ion lifetimes. We take advantage of the low outgassing at cryogenic temperatures to use standard printed circuit board assemblies for in-vacuum electronics. This reduces the technical effort compared to preparing ultra-high-vacuum-compatible electronics for room temperature operation^30^.

### Switching of trapping potentials

**Figure 5:**
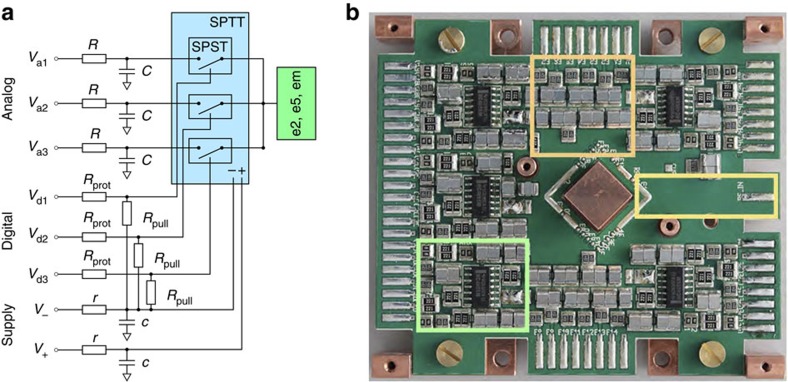
Cryogenic electronics. (**a**) Realization of a single-pole triple-throw (SPTT) switch for a switchable control electrode. *r* and *c* form low-pass filters for the supply lines of the CMOS switches (*V*_+_, *V*_−_), with a cutoff frequency of ≈14 kHz. *R* and *C* form low-pass filters for the input DC voltages (*V*_a_), with a cutoff frequency of ≈14 Hz. As usual in rf traps, the capacitors have the additional function of rf-grounding the control electrodes. *R*_prot_ and *R*_pull_ are over-current protection and pull-down resistors for the digital control lines of the single-pole single-throws (SPSTs). *V*_a_ is passed on to the electrode by closing the SPST with a digital high/low level through the control line *V*_d_. (**b**) Picture of the CEB. The circuitry relative to one of five switches is highlighted in green, a group of seven RC filters in orange and the position of the rf track (inside the printed circuit board) in yellow. The CEB is mounted on a copper structure for thermal contact to the cold plate.

To perform our experiments, we first trap an ion close to the position of the star in Fig. 4 and cancel stray electric fields to place the trap on the pseudo-potential null line. We then suddenly switch on an electric field directed along the trap axis. This is realized by switching to different voltages at electrodes e2, e8 and em, which are connected to the output of a single-pole triple-throw switch mounted on the Cryo-Electronics Board (CEB, Fig. 5b) and within 3 cm of the trap chip.

The CEB holds 30 low-pass filters for the analogue input lines, the fast switching electronics, and a track for guiding the trap rf drive from a quarter-wave helical resonator to the trap chip. For voltage switching we use a commercial complementary metal oxide semicondutor (CMOS) integrated circuit (CD74HC4066M, from Texas Instruments), which implements four bilateral single-pole single-throw switches. The additional circuitry to implement a single-pole triple-throw switch is shown in Fig. 5a. The CEB includes five such copies, one per switchable electrode. Measured parameters of this circuit are shown in Table 1. Resistors and capacitors are attached in parallel pairs to have a spare connection in the event of solder-point damage during cool-down. To ensure cryo-compatibility, all resistors are thin film and capacitors are from the Panasonic ECHU(X) series. Otherwise, the printed circuit board design and soldering techniques do not differ from those used to prepare standard non-vacuum boards.

One concern with the use of digital switches behind the filters is the spectrum of the electronic noise they introduce at the trap electrodes. To check whether this limits our heating rate, we measured this quantity with the CMOS integrated circuits unpowered with wire bridges connecting the switch inputs directly to the outputs. The heating rate in this case is the same as when making full use of the switches, suggesting they do not currently limit motional coherence.

The digital pulses for controlling the switches are produced by room-temperature electronics based on a multichannel delay/pulse generator (P400 from Highland Technology). Sequences of pulses are triggered by a single TTL line locked to the phase of the rf drive and generated by a field-programmable gate array, which we also use for the generation of laser-pulse sequences.

We generate the desired trapping potentials by applying voltages at the electrodes which we determine from analytical simulations^31^,^32^. First, we characterize the contribution of each electrode by calculating the electric potential generated above the trap chip when the electrode is set to 1 V and the rest are grounded. The total potential is given by the sum of individual contributions, weighted by the voltage applied to each electrode. We define the target overall trap potential in terms of its first and second-order derivatives, resulting in a system of equations which we solve to determine the electrode voltages. For the main trapping potential (*x*=0) we float all voltages. For the displaced trap (at *x*_d_), the non-switchable electrodes are fixed to the solutions obtained for the original potential and we solve only for the five switchable electrodes. The constraints in this case are given by the new trap position and axial frequency, which we desire to remain unchanged for these experiments. We also constrain the solutions to voltage spans of maximum 9 V, limited by the CMOS switches. Voltage offsets of <1 V at these five electrodes suffice to carry out our experiments (Table 2). Experimentally we find that the displaced solutions do not necessarily lie on the pseudo-potential axis, which we compensate with small correction fields.

### Cooling and detection

The experimental sequences are depicted in Fig. 1c,d. Ground-state cooling is achieved in two stages. The first involves Doppler cooling into the Lamb-Dicke regime by applying a laser 10 MHz detuned below the *S*_1/2_↔*P*_1/2_ transition at 397 nm for 500 μs. We then follow this with electromagnetically induced-transparency cooling, involving the application of two 397 nm laser fields polarized so that they drive simultaneously the *σ*^−^ and *π* transitions between the *S*_1/2_ and *P*_1/2_ Zeeman sub-levels. With suitable combinations of laser powers and frequency detunings, this allows us to cool the ion close to the ground state of motion^20^. In our set-up we reach a steady state at 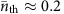 after 100 μs, limited by the heating rate of the ion, which is 1−2 kHz from the quantum ground state. The heating rate is higher than expected, but compatible with other systems given the large variation in reported results^33^. After cooling, the state is initialized to |↓〉 by optically pumping with a *σ*^−^-polarized resonant 397 nm beam. In all these pulses, 866 nm light is used to repump the population from *D*_3/2_ back to *P*_1/2_. Coherent operations between the *S*_1/2_ and *D*_5/2_ states are performed with a narrow-linewidth 729 nm laser. These couple the motional state to the spin system, which is subsequently read out by state-dependent fluorescence with resonant 397 and 866 nm light. In a detection time interval of 500 μs, we detect a mean number of 2 photons for an ion initially prepared in |↑〉, and 25 photons if the ion is prepared in |↓〉.

### Displaced thermal states

For a thermal state with average phonon number 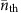 displaced by *α*, the occupation of the *n*^th^ number state is^34^


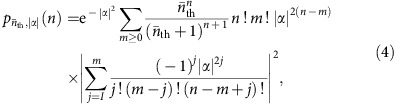


with *I*=max {0, *m*−*n*}.

### Light-atom interaction

The coupling between the internal electronic states and the ion's motion is described by the Hamiltonian^21^


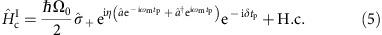


here, 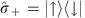 is the spin-flip operator producing transitions from |↓〉 to |↑〉 at the Rabi frequency Ω_0_/(2*π*), the Lamb-Dicke parameter *η*=*k*_*x*_*x*_0_ relates the motional ground-state wave packet size 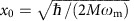 to the projection of the radiation wave vector ***k*** on the trap axis, *M* is the ion's mass and *δ* is the laser detuning from the carrier transition at frequency *ω*_0_. For integer values of *s*, laser light detuned by *δ*≈*sω*_m_ drives near-resonant transitions on the *s*^th^ motional sideband |↓〉 |*n*〉↔|↑〉 |*n*+*s*〉. The corresponding matrix element is proportional to 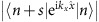 and its associated Rabi frequency can be evaluated analytically^18^:


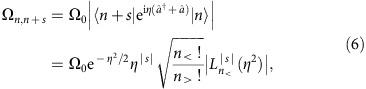


where, *n*_<_ (*n*_>_) stands for the lesser (greater) of *n*+*s* and *n*, and 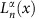 are generalized Laguerre polynomials.

### Dependence of deduced state sizes on Rabi frequencies

For the first set of experiments we carried out the sequence in Fig. 1c and obtained the results in Fig. 2. Here we focus on one of the consequences of the slow drifts in laser power, which are not included in the model used for theoretical calculations.

From a first fit of a non-displaced state we obtain Ω_0_/(2*π*)=181(1) kHz and *Γ*=2.2(3) ms^−1^, using equation (3) with |*α*|=0 and 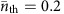. From subsequent fits for each value of Δ*t* we obtain the coherent-state sizes plotted in Fig. 2a. Fitting these to equation (1), we determine *α*_0_=5.11(1) and *ω*_m_/(2*π*)=2.3505(6) MHz. However, we note that over the time required to take this data the Rabi frequency varies over a range *δ*Ω_0_/(2*π*)≈±3.2 kHz. The value of |*α*| determined from fits is strongly correlated to the Rabi frequency used, which accounts for the fluctuations observed in Fig. 2a. To illustrate this we repeat the fits fixing the Rabi frequency to the limiting values Ω_0_±*δ*Ω_0_ and floating *α*_0_ and *ω*_m_. This yields *α*_0_=5.00(1) for Ω_0_+*δ*Ω_0_, and *α*_0_=5.22(1) for Ω_0_−*δ*Ω_0_, with *ω*_m_/(2*π*)=2.3505(6) MHz in both cases. The boundaries of the shaded area in Fig. 2a are obtained by inserting these numbers into equation (1).

### Motional-state-dependent AC Stark shifts

**Figure 6:**
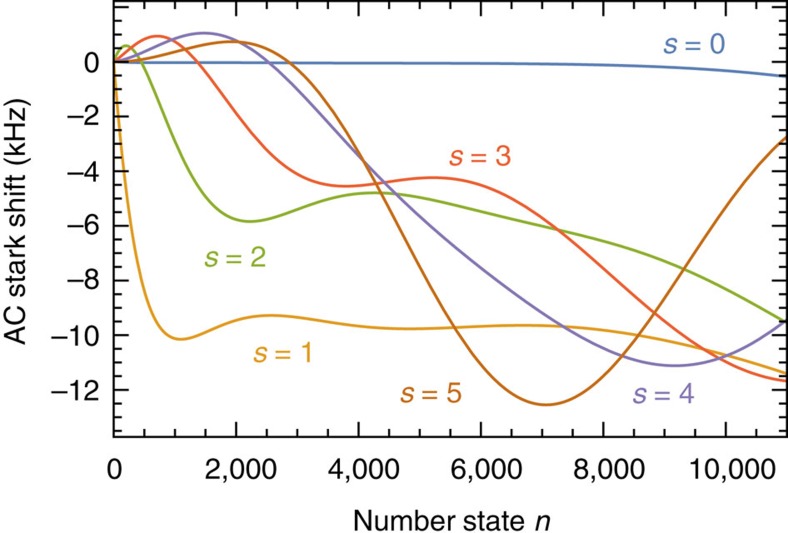
AC Stark shifts. Energy level corrections due to off-resonant coupling to motional sidebands other than the one driven, as well as to the carrier and sidebands of the |↓〉↔| *L*=2, *J*=5/2, *M*_*J*_=3/2〉 transition at our 3.83 G magnetic field when the laser is resonant with the transition for *n*=0.

The model used for calculating the expected *P*_↓_ in our experiment (Fig. 3) includes a detuning from the driven motional sideband as well as an AC Stark shift due to off-resonant coupling to transitions other than the one probed. The latter is given by


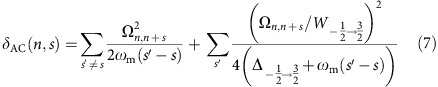


and plotted in Fig. 6 for our experimental parameters. The first term arises from coupling to sidebands of the spin transition, while the second is due to the fact that the 729 nm laser drives off-resonantly the secondary transition |↓〉↔|*L*=2, *J*=5/2, *M*_*J*_=3/2〉. The coupling strength to the secondary transition is reduced relative to that of the resonantly driven transition by a factor 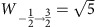. At our magnetic field of ≈3.83 G, the frequency gap between both carrier transitions is 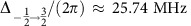. This is close to an integer number of times the motional frequency, causing the *s*=4 (5) sideband of the main transition to be almost resonant with the *s*=−7 (−6) sideband of the secondary transition, and therefore strongly AC Stark shifting the |↓〉 state. For a total detuning *δ*_tot_(*n*, *s*)=*δ*_off_(*s*)+*δ*_AC_(*n*, *s*), equation (3) can be re-written as


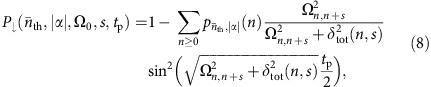


where we have ignored the exponential-decay term due to decoherence, since our probe times go up to 80 μs and we found previously *Γ*≈2 ms^−1^.

### Light-matter-interaction data analysis

For this experiment we carry out the sequence in Fig. 1d. The results are shown in Fig. 3a. To analyse the data and compare it with the theoretical model, we first calibrate the displacements with the data obtained for the carrier (*s*=0). We fit the measurement results for each value of *x*_d_ to equation (8) while fixing Ω_0_ and 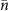 to values determined previously by driving Rabi oscillations on the *s*=0 and *s*=1 transitions, respectively. This yields a coherent-state size which we convert into displacements according to *α*_0_=*x*_d_/(2*x*_0_). The calibrated displacements deviate by <50 μm from the nominal displacements calculated as described in section 4.2. For the rest of the sidebands we use the calibrated displacements and fit the measurement data to find Ω_0_ and *δ*_off_ for each individual sideband. The best fit parameters are given in Table 3 and yield the results in Fig. 3b,c.

**Figure 7:**
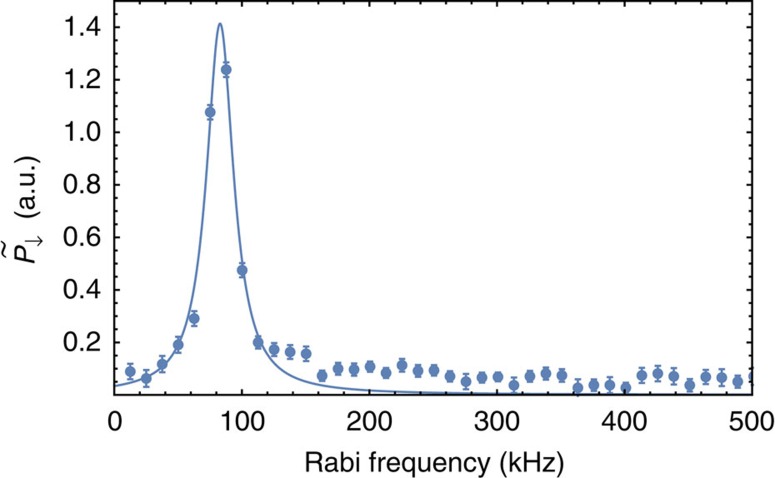
Rabi-frequency spectrum. The data points and curve are obtained as explained in the text. This plot is for the carrier transition (*s*=0) and a displacement *x*_d_≈660 nm (*α*_0_≈44).

To analyse the evolution of the mean Rabi frequency for a given driven sideband *s* and as a function of the state size (Fig. 3c), we calculate the discrete Fourier transform of the measured *P*_↓_(*t*_p_) and propagate the shot-noise uncertainties to the frequency domain according to ref. 35. The number-state distribution 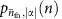 for low values of 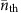 is narrow compared with the features which result from equation (6), so the frequency spectrum shows a single peak at the mean Rabi frequency (Fig. 7). We fit these to symmetric Lorentzian functions, from which we determine the center frequencies given in the plots. We compare this to the theoretical mean Rabi frequency for a displaced thermal state, given by


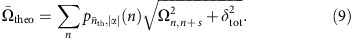


The separation between minima in mean Rabi frequency depends on the radiation wavelength *λ*. For large values of *n*, the Laguerre polynomials in equation (6) can be approximated by Bessel functions *J*_*α*_ as^36^


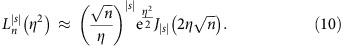


The zeros in the right-hand side correspond to zeros of the Bessel functions, whose separation tends to *π* for large arguments. This implies that the separation between minima in mean Rabi frequencies tends to *λ*/(2cos*θ*) for large values of *x*_d_.

## Additional information

**How to cite this article:** Alonso, J. *et al.* Generation of large coherent states by bang–bang control of a trapped-ion oscillator. *Nat. Commun.* 7:11243 doi: 10.1038/ncomms11243 (2016).

## Figures and Tables

**Table 1:** Switch characteristics.

	**300 K**	**4 K**
Off resistance (MΩ)	>10	>10
On resistance (Ω)	12	3.5
Power consumption (mW)	8	10
Crosstalk (dB)	−50	−50
Rise/fall time (ns)	13	<5

Summary of the measured performance of the switching electronics at 300 and 4 K. Off and on resistance are measured directly at the switch. The power consumption was measured at a switching rate of 1 MHz. The crosstalk indicated is the fraction of power from the digital side leaking to the analogue output, and is measured around 1.5 MHz. The rise/fall times are given for the 10–90% transition of a 9-V step.

**Table 2:** Voltages at switchable electrodes.

**Electrode**	**Trap at** ***x*****=0**	**Trap at** ***x***_**d**_**≈1.5 μm**	
e2l	−0.5016		−0.5218
e2r	5.8781		5.6050
e8l	0.7885		0.4343
e8r	2.8146		3.4488
em	0.4962		0.4868

Voltages (in volts) applied to the trap electrodes for displacing the trap potential by ≈1.5 μm, corresponding to the largest displacements in these experiments. The voltages are generated by commercial digital-to-analogue converter chips with specified stability and resolution of ≈1.2 mV (AD5371 from Analogue Devices).

**Table 3:** Light-matter-interaction fits.

***s***	**Ω**_**0**_**/(2*****π*****) (kHz)**	***δ***_**off**_**/(2*****π*****) (kHz)**
0	205	0
1	211	4
2	218	−4
3	224	−12
4	226	−23
5	226	−32

Fit results for the light-matter-interaction experiment. The probe-pulse frequencies for sidebands *s*=0–2 were pre-calibrated with non-displaced states. Higher order sidebands are too weak for a direct measurement with non-displaced states, so probe-pulse frequencies for *s*>2 where estimated from the lower sideband frequencies. This explains the large systematic shifts in *δ*_off_ resulting from the fits.
